# Visceral Leishmaniasis in Southern Brazil: an overview of current epidemiology

**DOI:** 10.1590/0037-8682-0240-2025

**Published:** 2026-03-02

**Authors:** Bibiana Paula Dambrós, Amabilli de Souza Rosar, Patricia Hermes Stoco, Patricia Flávia Quaresma, Edmundo Carlos Grisard, Mario Steindel

**Affiliations:** 1 Instituto Federal de Santa Catarina, Campo Duna, Garopaba, SC, Brasil.; 2 Universidade Federal de Santa Catarina, Departamento de Microbiologia, Imunologia e Parasitologia, Laboratório de Protozoologia, Florianópolis, SC, Brasil.

**Keywords:** Visceral leishmaniasis, Canine visceral leishmaniasis, Sandflies fauna, Epidemiology, Leishmania infantum

## Abstract

Visceral leishmaniasis (VL), caused by the protozoan parasite *Leishmania* (*Leishmania*) *infantum*, is transmitted in Brazil through the bites of female phlebotomine sandflies. Until 2008, no cases of autochthonous VL transmission were reported in the southern Brazilian states of Paraná (PR), Santa Catarina (SC), and Rio Grande do Sul (RS). The first case of canine VL (CVL) in RS was reported in 2008, followed by that of human VL (HVL) in 2009. Since then, CVL and HVL cases have emerged in 12 and five additional municipalities in the RS, respectively. In SC, CVL was first reported in Santa Catarina Island in 2010 and is now present in 44 of 89 localities. By 2014, CVL had appeared in western SC, with new cases reported in the midwestern and southern areas in 2021and 2024, respectively. Between 2017 and 2022, five autochthonous HVL cases were diagnosed in Florianópolis. In the PR, CVL was first reported in Foz do Iguaçu in 2012, followed by HVL in 2015. Although *Lutzomyia longipalpis*, the primary vector of *L. infantum* in Brazil, has been found in certain municipalities of RS and PR, it has not been reported in other endemic or vulnerable areas of RS and SC. This suggests that other sandfly species may also contribute to disease transmission. This highlights the urgent need for research on the diversity of hosts, vectors, and parasite strains, along with enhanced epidemiological surveillance to clarify the transmission dynamics of VL in these regions.

## INTRODUCTION

Visceral leishmaniasis (VL) is a potentially fatal disease caused by the protozoan parasites *Leishmania donovani* and *Leishmania infantum*, affecting 83 countries and territories, resulting in 50,000-90,000 cases per year^1^ ^,^ ^2^. Transmitted through the bite of infected female sandflies of the genus *Lutzomyia* in the Americas, symptoms include fever, weight loss, fatigue, weakness, loss of appetite, enlarged liver and spleen, anemia, and swollen lymph nodes^1^. This neglected and socially determined tropical disease mainly affects vulnerable populations and is one of the most significant infectious diseases worldwide^1^. 

Human VL (HVL) cases have been reported in Argentina, Bolivia, Brazil, Colombia, Costa Rica, El Salvador, Guatemala, Honduras, Mexico, Nicaragua, Paraguay, Uruguay, and Venezuela. These countries report approximately 3,500 cases per year, ranging from 7% to 9%. Over 90% of cases occur in Brazil^3^, which has one of the highest rates of canine VL (CVL) worldwide^4^. Thus, parasite circulation through humans, vectors, and mammals potentially allows distinct genotypes or adaptations to local vectors^5^ ^-^ ^7^.

Historically, in impoverished endemic rural areas, CVL and HVL have progressively spread to the mid- and southwestern regions of Brazil, establishing an urban transmission cycle in which CVL precedes human cases in 23 of 26 states, including the Federal District^8^ ^,^ ^9^. In this review, we examined the expansion of CVL and HVL in the southern Brazilian states of Paraná (PR), Santa Catarina (SC), and Rio Grande do Sul (RS), as well as their epidemiological aspects and prophylactic strategies.

## METHODS

To evaluate the status of autochthonous canine and human VL, as well as the phlebotomine fauna and epidemiology of VL in southern Brazil, a comprehensive literature search was conducted using the following primary databases: PubMed, SciELO, and SCOPUS, covering the period from January 2008 to December 2024. The following terms were used to retrieve articles: “Canine OR Human Visceral Leishmaniasis AND Rio Grande do Sul OR Santa Catarina OR Paraná,” “*Lutzomyia* OR Phlebotomine OR Sandfly AND Rio Grande do Sul OR Santa Catarina OR Paraná,” “Leishmaniasis OR Epidemiology AND Rio Grande do Sul OR Santa Catarina OR Paraná,” and “Visceral Leishmaniasis OR Epidemiology AND Southern Brazil.” 

This review adhered to the guidelines outlined in the Preferred Reporting Items for Systematic Reviews and Meta-Analyses statement (PRISMA), which can be accessed at http://www.prisma-statement.org. The inclusion criteria were as follows: (1) articles written in English or Portuguese, (2) free full access or availability through Periódicos CAPES, and (3) publication dates and data collection from 2008 to 2024. The exclusion criteria were as follows: (1) reviews, theses, dissertations, monographs, editorials, letters to the editor, forums, comments, author corrections, and books, (2) studies with overlapping or duplicate data, and (3) abstract-only papers.

**FIGURE 1:**
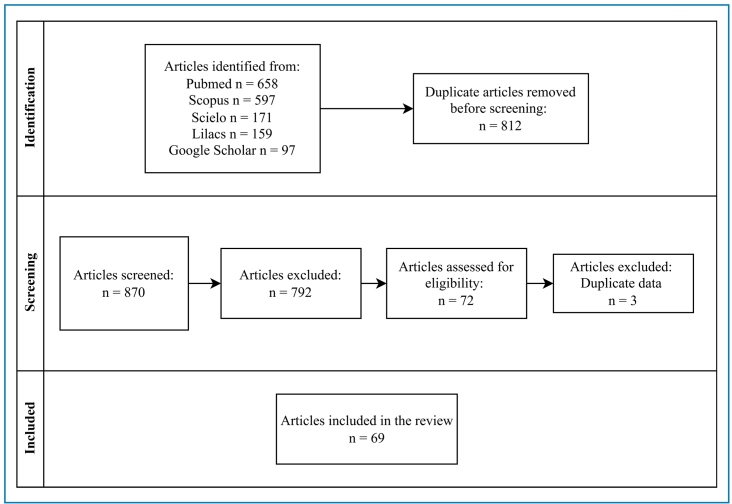
Flowchart of article selection.

Only full papers in English and Portuguese were reviewed, and only those containing detailed information were retrieved and analyzed according to the eligibility criteria. To minimize potential bias in the literature search, the titles and abstracts of the selected articles were examined by two independent reviewers based on predefined inclusion criteria (Figure 1).

**FIGURE 2:**
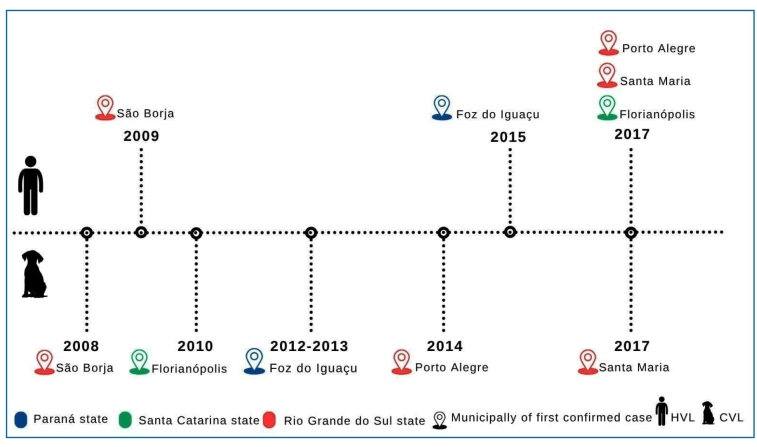
Timeline of autochthonous canine **(CVL)** and human **(HVL)** visceral leishmaniasis in southern Brazilian states of Paraná (PR), Santa Catarina (SC), and Rio Grande do Sul (RS).

**FIGURE 3:**
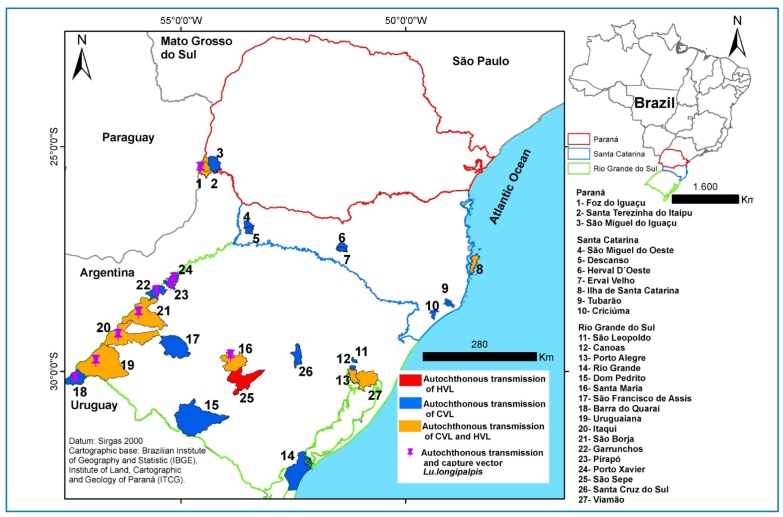
Distribution of autochthonous cases of canine visceral leishmaniasis **(CVL)** and human visceral leishmaniasis **(HVL)** and the presence of *Lutzomyia longipalpis* in the states of Paraná (PR), Santa Catarina (SC), and Rio Grande do Sul (RS) between 2008 to 2024.

Additionally, data were retrieved from the World Health Organization (WHO), Pan American Health Organization (PAHO), Brazilian Health Authority databases (SINAN and DATASUS), and technical publications from the RS, SC, and PR state health authorities. Raw data were manually curated to include data exclusively related to southern Brazilian States and then organized in Excel spreadsheets to allow comparative analysis. Figure 1 and Figure 2 were generated using Canva (https://www.canva.com/pt_br/), and Figure 3 was generated using QGIS Desktop 3.40.10.

## CANINE AND HUMAN VISCERAL LEISHMANIASIS

HVL has been documented in Brazil since 1913; however, *L. infantum* was likely introduced to Brazil by Portuguese or Spanish colonizers in the northeastern region, where susceptible vectors facilitated parasite transmission^10^. Since then, primary routes for *L. infantum* dispersion in central and southern Brazil have been proposed.

Retrospective analyses have pointed to the Bolivia-Brazil route of introduction, associated with population/animal displacement from Bolivia to the neighboring states of Mato Grosso and Mato Grosso do Sul in Brazil, and possibly São Paulo, during the construction of the Bolivia-Brazil gas pipeline (1998-2005)^11^.

Microsatellite analyses have indicated two possible introduction routes. The Paraguay-Brazil route, linked to the introduction of VL from urban transmission in Paraguay into the Brazilian states of Mato Grosso do Sul and Paraná, particularly around the triple border area (Argentina-Paraguay-Brazil) around 2012; and the introduction of a distinct genetic cluster of *L. infantum* strains in western Santa Catarina, closer to the Argentinian border, that expanded towards southern Paraná state around 2013^11^.

As the introduction of VL into the RS was associated with CVL and HVL cases reported in Posadas, Argentina, in 2006^12^ ^,^ ^13^, we cannot rule out the expansion of VL within the Brazilian territory, since the country has one of the highest prevalence rates in the Americas.

The emergence of VL in the southern states is a recent phenomenon, as only a few imported human cases were reported before 2007^14^ ^-^ ^17^. Since then, a typical pattern of disease expansion has been observed, with increasing reports of autochthonous CVL, followed by HVL (Figure 2).

Autochthonous cases of CVL in RS were initially reported in São Borja in 2008, close to the Brazil-Argentina border, where *Lu. longipalpis* was identified as the primary vector, highlighting the vulnerability of these areas^18^ ^,^ ^19^. In the following year, autochthonous HVL cases were reported in the same municipality^18^. In 2010, autochthonous CVL cases were reported in the metropolitan area of Porto Alegre, the state capital, located approximately 585 km east of São Borja^20^ ^-^ ^22^. Between January and July 2014, the CVL seroprevalence was 4.0% in Porto Alegre, reaching 5.6% in Canoas and 4.6% in São Leopoldo neighboring cities^20^. Between 2016 and 2021, 766 CVL and 20 HVL autochthonous cases were reported in Porto Alegre^21^, where *Lu. longipalpis* has not been reported^23^. In 2010, an autochthonous case of CVL was reported for the first time in the non-endemic municipality of Santa Maria in the central region of the state^24^. Almost simultaneously, several autochthonous CVL and two cases of HVL were recorded between 2017-2021 in Santa Maria, in the central region of RS, where the presence of *Lu. longipalpis* was confirmed^25^ ^-^ ^27^.

Between 2009 and 2023, 52 autochthonous HVL cases were documented in RS, with seven fatal cases in Porto Alegre and one in Santa Maria^26^ ^,^ ^28^, indicating the spread of VL and its associated morbidity and mortality in the region. According to the RS State Health Authority, the active transmission of *L. infantum* was recorded in 12 municipalities (Figure 3), 67% of which shared borders with Argentina and Uruguay^28^.

By 2023, autochthonous HVL in southern Brazilian states had an annual average rate of 0.67 cases for SC, 1.0 for PR, and 4.67 for RS, encompassing low transmission risk areas^29^ (Table 1). However, a comparative analysis of data obtained from the Brazilian Ministry of Health, State Health authorities, and scientific literature revealed some discrepancies. For instance, municipalities such as Castro and Umuarama in PR, Imaruí and Joinville in SC, and Cachoeira do Sul, Capão da Canoa, Nova Petrópolis, Rio Grande, and São Sepé in RS were classified as areas of autochthonous HVL transmission despite a lack of evidence^28^ ^,^ ^30^ ^,^ ^31^. Conflicting epidemiological reports must be properly addressed, as the accuracy of such data depends primarily on specific diagnoses, directly impacting treatment, decision-making processes, and the effectiveness of educational campaigns, demanding collaboration between health authorities, researchers, and the public. 

**TABLE 1:** Number of reported and confirmed cases of human visceral leishmaniasis in the states of Paraná, Santa Catarina, and Rio Grande do Sul between 2009 to 2023, according to DATASUS.

State	Origin (municipality of infection)	Number of cases		Deaths
		Reported	Confirmed	
**Paraná**	Foz do Iguaçu	24	24	6
	Maringá	2	0	0
	Santa Terezinha de Itaipú	2	2	0
	Alto Paraiso	1	0	0
	Castro	1	0	0
	Curitiba	1	0	0
	Ivate	1	0	0
	Londrina	1	0	0
	Terra Rica	1	0	1
	Umuarama	1	0	0
**Santa Catarina**	Florianópolis	5	5	0
	Imaruí	1	0	0
	Joinville	1	0	0
**Rio Grande do Sul**	Porto Alegre	25	25	7
	São Borja	14	14	0
	Uruguaiana	6	6	0
	Itaqui	4	4	0
	Viamão	4	0	0
	Santa Maria	3	3	1
	Cachoeira do Sul	1	0	0
	Capão da Canoa	1	0	0
	Nova Petrópolis	1	0	0
	Rio Grande	1	0	0
	São Sepe	1	0	0
**Total**		**103**	**83 (80.6%)**	**15 (14.5%)**

A retrospective analysis of the evolution of CVL in the state of PR illustrated the challenges associated with diagnosis, epidemiological assessment, and decision-making for the control of disease transmission. A seroepidemiological survey of CVL was conducted in the municipalities of Foz do Iguaçu and Santa Terezinha de Itaipu between 2014 and 2015, and 1,129 dogs were tested^32^. The seroprevalence of CVL was 23.8% in the rural areas of Foz do Iguaçu and 4.7% in the neighboring city of Santa Terezinha de Itaipu^32^. Between 2015 and 2020, a survey of CVL conducted on 12,205 dogs in Foz do Iguaçu revealed a seroprevalence of 37.94%, indicating a heightened risk for HVL^33^. 

By 2015, the first autochthonous case of HVL was diagnosed^34^. Between 2015 and 2022, these municipalities, located on the triple border between Argentina and Paraguay, reported 26 autochthonous HVL cases, resulting in six fatalities^29^. This highlights the potential of VL to spread across neighboring municipalities and countries. 

The emergence of CVL in SC began in 2010 in the neighborhoods of Canto dos Araçás and Lagoa da Conceição on Santa Catarina Island (Florianópolis), located on the eastern Atlantic border^17^ ^,^ ^35^. According to data from the Zoonosis Control Center in Florianópolis, the percentage of euthanasia cases was 57.35% between 2010 and 2019. In contrast, from 2020 to 2022, the percentage decreases to 28%. In 2013 and 2015, CVL surveys were conducted in São Miguel do Oeste and Descanso in the western region of the state^36^ and in Erval Velho and Herval d’Oeste in the midwestern region^37^. Based on serological and PCR testing, these surveys revealed prevalences of 19.0% and 7.14% in the western and midwestern regions, respectively. Following the canonical spreading pattern, three autochthonous HVL cases were reported in Florianópolis in 2017, with two additional cases each in 2020 and 2023^31^ ^,^ ^38^.

An overall seroprevalence of 0.6% for CVL was estimated among 1,227 dogs sampled across six mesoregions of SC using DPP and ELISA tests^39^. Positive cases were primarily concentrated in the eastern region of the state, with 43% of these cases being asymptomatic and 57% exhibiting polysymptomatic characteristics. Later, a survey of *L. infantum* infection based on PCR analysis of blood samples from dogs sheltered at the Zoonosis Control Centers in the cities of Tubarão (77 dogs) and Criciúma (30 dogs), located in southeastern SC, revealed positivity rates of 16.9% and 23.5%, respectively^40^. Notably, 85% of the dogs were asymptomatic. Another study in Tubarão reported a PCR positivity rate of 4.2% for *L. infantum* in conjunctival swabs from 47 sheltered dogs^41^. Taken together, these findings confirm the transmission of *L. infantum* in SC, not only in Florianópolis, but also in the southern, midwestern, and western regions. This indicates that the seroprevalence rates may vary considerably owing to the different sampling and diagnostic methods employed. According to the SC Health Authority, between 2019 and 2020, 50 of 295 municipalities reported CVL cases, mostly non-autochthonous^42^, whereas Florianópolis recorded 1,058 CVL cases in 11 out of 18 districts by 2024^43^, representing an increased risk of VL transmission to humans.

## SANDFLY FAUNA

Phlebotomine sand flies are blood-feeding insects and vectors of *Leishmania* parasites, the causative agents of leishmaniasis. Sand flies are found in tropical and temperate regions worldwide, and the majority of medically important species belong to the genus *Phlebotomus* in the Old World and *Lutzomyia* in the New World^44^. Thus, the assessment of local sandfly fauna, the prevalence of *Leishmania* spp. infection, and the vectorial competence of these species is crucial for understanding the epidemiology of VL. Xenodiagnosis is a classical parasitological technique used to determine and quantify the infectiousness of a specific host to a blood-sucking insect species, and provides conclusive data to distinguish infectious from noninfectious^45^. However, this method is labor-intensive, complex, and time-consuming, and requires the establishment of sandfly colonies, trained personnel, and research facilities.

The complex interactions between mammalian-infective *Leishmania* species or genotypes and sand flies may influence transmission dynamics and require multivariate analysis^46^. In the context of VL, such studies are of major relevance to endemic or vulnerable transmission areas where the primary vector *Lu. longipalpis* and *Lu. cruzi* have not yet been reported. Other sandfly species, such as *Lu gaminarai*, *Mi. migonei*, *Ny. neivai*, *Pi. fischeri*, and *Lu. evansi* have also been implicated in the transmission of *L. infantum* in Brazil^14^ ^,^ ^47^ ^-^ ^51^.

The state of Paraná was considered free of VL transmission until 2012, with neither CVL, HVL, nor *Lu. longipalpis* reported there^52^ ^,^ ^53^. *Lu. longipalpis* was sampled for the first time in Foz do Iguaçu in 2012, when the city was declared an area at risk for the disease^54^. An entomological survey conducted in Foz do Iguaçu and Santa Terezinha de Itaipu between 2014 and 2015 revealed that *Lu. longipalpis* was the predominant species, accounting for 71.78% of the 3,543 sandflies captured^55^. Other species, such as *Evandromyia edwardsi*, *Expapillata firmatoi*, *Micropygomyia ferreirana*, and *Pi. christenseni* documented for the first time in this region, may also be involved in *L. infantum* transmission^55^. 

A summary of the phlebotomine sandfly fauna in vulnerable and endemic areas of VL in southern Brazil is presented in Table 2.

**TABLE 2:** Records of sandfly species in endemic areas of visceral leishmaniasis in the states of Paraná, Santa Catarina, and Rio Grande do Sul.

State	Municipality	Sandfly species	References
**Paraná**	Foz do Iguaçu and Santa Terezinha de Itaipu	*Lu. longipalpis*, *Ny. whitmani*, *Ny. intermedia*, *Ny. neivai, Mi. quinquefer*, *Mi. migonei*, *Pi. pessoai*, *Pi. monticola*, *Pi. fischeri, Pi. christenseni, Brumptomyia brumpti*, *Martinsmyia alfabética*, *Ev. cortelezzii, Ev. edwardsi, Evandromyia* sp.*, Pa. shannoni, Expapillata firmatoi, Micropygomyia ferreirana,* Phlebotominae sp.	^5^ ^,^ ^54^ ^,^ ^55^
**Santa Catarina**	Florianópolis	*Pi. fischeri, Mi. migonei, Ny. neivai, Brumptomyia* sp.	^14^ ^,^ ^42^
	Joaçaba, Herval d'Oeste, Ouro, Lacerdópolis and Erval Velho	*Brumptomyia cunhai, Br. guimaraesi, Br. mangabeirai, Brumptomyia* sp*., Mi. migonei, Ny. neivai, Pi. fischeri, Ps. lanei, Ev. edwardsi, Martinsmyia alfabetica*	^60^
	Tubarão	*Ny. neivai*, *Mi. migonei*, *Pi. fischeri*, *Ev. edwardsi*, *Brumptomyia* sp*.*	^41^
**Rio Grande do Sul**	São Borja, Barra do Quaraí, Garruchos, Pirapó, Porto Xavier, Uruguaiana, Itaqui	*Lu. longipalpis*	^18^
	Porto Alegre	*Ps. lanei, Brumptomyia* sp., *Mi. migonei, Pi. fischeri, Ny. neivai, Lutzomyia gaminarai*	^21^ ^,^ ^23^ ^,^ ^50^
	Estrela	*Brumptomyia cunhai, B. nitzulescui, Ev. edwardsi, Pi. fischeri, Ps. lanei, Mi. migonei, L. misionensis, Ny. neivai, L. pascalei*	^48^
	Santa Maria	*Lu. longipalpis, Mi. migonei, Pi. fischeri, Ps. lanei, Brumptomyia* sp.	^26^ ^,^ ^27^

In 2006, the first case of VL was identified in Argentina’s province of Misiones, where several cases of CVL and one case of HVL were reported in Posadas along with the presence of *Lu. longipalpis*. This represents the southernmost transmission of *L. infantum* in Latin America^12^. Shortly after, *Lu. longipalpis* was also reported in Salto and Bella Unión-Cuarein in Uruguay^56^, where CVL cases were confirmed in 2015^57^ ^,^ ^58^, indicating the ongoing cross-border spread of VL and underscoring the necessity for both national and international surveillance efforts^55^ ^,^ ^56^ ^,^ ^58^.

Since 1990, Santa Catarina has reported the autochthonous transmission of human cutaneous leishmaniasis in the western region. Entomological surveys conducted in the northeastern and midwestern regions of SC did not record the presence of *Lu. longipalpis* ^59^ ^-^ ^61^. Since 2011, entomological surveillance conducted by the Zoonosis Control Centre in Florianópolis has revealed the presence of *Brumptomyia* sp., *Pi. fischeri*, *Mi. migonei*, *Ny. neivai*, *Ev. edwardsi*, *Lu. tupynambai*, and *Lu. firmatoi*. Natural infection assessed by PCR confirmed the presence of *L. infantum* DNA in 1.6% of *Ny. neivai*, as well as *Leishmania* spp. in 0.6% of *Pi. fischeri* and 0.3% of *Lu. migonei* ^14^. In a more recent study, *L. infantum* was confirmed in 0.4% *Ny. neivai* in Tubarão, 150 km south of Florianópolis, accounting for 85.8% of the sandflies captured^41^. In this area, a CVL prevalence of 4.2% was also confirmed. 

Approximately 50% of the 52 autochthonous HVL cases in RS occurred in areas where *Lu. longipalpis* has not been reported, including in the capital city of Porto Alegre, where sandfly fauna were investigated between 2014 and 2015 for *L. infantum* infection using PCR. In the Agronomia district, located 2.5 km from the Jardim Carvalho district, *Pi. fischeri* tested positive for *L. infantum* ^23^. Meanwhile, in the Morro Santana and Jardim Carvalho districts, which are 3.5 km apart, *Lu. gaminarai*, *Pi. fischeri*, and *Mi. migonei* also tested positive^50^. These findings in a restricted, vulnerable urban area bordering a forested region highlight the necessity for ongoing biological and epidemiological research to understand the dynamics of VL transmission, which may differ between endemic areas. Understanding these unique transmission patterns is crucial for developing targeted interventions and control strategies tailored to specific challenges faced in such environments.

The high prevalence of CVL and HVL in Porto Alegre (RS) and Florianópolis (SC), where *Lu. longipalpis* has not been reported, suggests that other species, such as *Ny. neivai, Mi. migonei*, *Pi. fischeri*, and *Lu. gaminarai*, which have been found naturally infected with *L. infantum,* may also play a role in *L. infantum* transmission in these regions.

## DIAGNOSIS OF CVL

The clinical diagnosis of CVL relies on clinical signs and clinicopathological abnormalities; however, a definitive diagnosis requires serological, parasitological, and molecular laboratory tests^62^. The Brazilian Ministry of Health recommends two serological tests based on different methodologies: an initial immunochromatographic dual-path platform (TR-DPP) (Bio-Manguinhos, Fiocruz, Brazil) and an ELISA assay for confirmation^63^. An indirect immunofluorescence assay (IFA) using standardized antigens and serum dilutions of at least³ 1:160 represents an alternative diagnostic assay^63^.

The microscopic examination of stained smears from popliteal lymph nodes or bone marrow aspirates and *in vitro* culture of these aspirates are useful parasitological techniques; however, histopathological and immunohistochemical tests have limited specificity and sensitivity^63^.

Molecular diagnosis using PCR offers high specificity and sensitivity, even in the early stages of infection, and can detect asymptomatic infections^64^ ^,^ ^65^. PCR techniques targeting *L. infantum* sequences such as kinetoplast DNA minicircles (kDNA), 18S rRNA, HSP70, and hypothetical proteins (Linj31) facilitate accurate diagnosis, parasite load monitoring, treatment evaluation, and assessment of vector infectiousness^66^ ^-^ ^69^.

Diagnostic methods vary among the southern states. According to the state and municipal healthcare authorities of PR, SC, and RS, DPP and ELISA are currently used in CVL surveys in all states. In SC and RS, standard immunological techniques (DPP and ELISA) are used to diagnose HVL and CVL, as recommended by the Brazilian Ministry of Health. In PR, where the Instituto Carlos Chagas (ICC/Fiocruz) is the state reference laboratory, serological, molecular, and parasitological methods are employed as confirmatory/complementary diagnostic tools for both HVL and CVL. 

## PROPHYLATIC AND CONTROL MEASURES

For over five decades, the Brazilian Ministry of Health has recommended dog culling as the primary public health measure for controlling CVL^4^ ^,^ ^62^ ^,^ ^70^. However, this policy has led to societal conflict, and numerous studies have shown that dog culling is ineffective as a standalone strategy to control CVL transmission^4^ ^,^ ^70^ ^,^ ^71^, prompting calls for new and more effective approaches^62^. Combined strategies, such as the use of repellents, vaccination, and treatment, may work at the individual level. However, public policies with community engagement may provide more humane and effective methods for managing the impact of VL on human and animal populations. 

Since 2016, the Brazilian government has authorized the use of miltefosine (Hexadecylphosphocholine, Milteforan®) for the treatment of CVL, representing the first therapeutic alternative to euthanasia for infected animals^72^. Treatment depends on the disease stage and can be achieved by the administration of miltefosine alone or in combination with domperidone or allopurinol. While miltefosine can initially improve symptoms and reduce the parasitic load, monotherapy yields unsatisfactory results because many animals experience relapse, which increases the risk of disease transmission^73^. To mitigate this, veterinarians have also used immunomodulators, including allopurinol, domperidone, and the Leish-Tec® vaccine when it was still available^73^ ^-^ ^75^, as recommended by Brasileish and Conselho Federal de Medicina Veterinária (CFMV)^63^ ^,^ ^76^. Therefore, the continuous development of comprehensive treatment protocols that incorporate immunotherapy, along with careful monitoring and supportive care, is essential for managing CVL and reducing the risk of transmission to humans^73^ ^,^ ^74^.

Although treatment with miltefosine and associated therapies can help reduce the parasite burden and improve the health of VL-infected dogs^73^, cases of parasitological cure have not been documented. Thus, a significant issue remains as to whether treated animals can act as reservoirs of *L. infantum*, which severely affects transmission control. Therefore, neither miltefosine therapy nor prophylactic or immunotherapeutic vaccination for CVL is a public health measure in Brazil^77^. 

Consequently, the responsibility and costs associated with the vaccination or treatment of infected dogs fall solely on their owners, demanding that dog owners take responsibility for their pets’ health and highlighting the importance of individual accountability in managing CVL cases^63^ ^,^ ^76^.

It is well established that dogs play a pivotal role as reservoirs to maintain the urban transmission cycle of VL in Latin American and Mediterranean countries^78^ ^,^ ^79^. The proximity of *L. infantum*-infected dogs to humans, parasite genetic plasticity, and the variety of sandfly species significantly influence disease dynamics, requiring control and prevention strategies. Since 2020, the CFMV has implemented mandatory reporting of positive or suspected CVL cases in Brazil, recommending complementary preventive and control measures, such as vaccination and the use of insecticide-impregnated collars^76^. In southern Brazil, key control measures include the euthanasia of infected dogs, seroepidemiological surveys in transmission areas, establishment of municipal zoonosis control centers, and urban/peridomiciliary cleaning to eliminate potential breeding sites for sandflies. The municipality of Florianópolis (SC) has offered free treatment to VL-infected dogs from low-income owners since 2022^80^, and the SC State Health Authority has implemented mandatory reporting of CVL since 2024^81^.

Finally, it is worth noting that various sylvatic and domestic mammals of the orders Carnivora, Marsupialia, and Rodentia act as primary reservoirs of *L. infantum*, thereby sustaining the zoonotic transmission cycle^82^, which has been understudied across a wide geographical area.

## FINAL REMARKS

The geographic expansion of VL in southern Brazil is an undeniable reality that emphasizes the need for continuous monitoring and improved intervention strategies towards risk management in both endemic and vulnerable areas. However, it is crucial to consider that inadequate entomological and medical surveillance, as well as non-standardized human or animal testing, may lead to underreporting of both CVL and HVL, which can significantly affect our understanding of the disease’s epidemiology in southern Brazil.

Additionally, the border effect, along with increased domestic and international migration and cargo transport in recent decades, raises concerns regarding the potential spread of VL. These factors necessitate the effective control of the movement of infected animals to mitigate the risk of transmission. Comprehensive strategies focusing on surveillance, public awareness, and strict regulations regarding animal transport are essential to address the challenges posed by VL in this region.

While *Lu. longipalpis* is a key player in the epidemiology of VL throughout Brazil, other sandfly species, often regarded as secondary vectors, may act as competent or primary vectors of *L. infantum* in areas where *Lu. longipalpis* has not yet been reported. This is supported by the high seroprevalence of CVL in vulnerable and formerly non-endemic areas. These findings suggest the need for further research on the roles of alternative vectors and their potential impact on the transmission dynamics of leishmaniasis, as well as the importance of adapting control measures to address the specific risks associated with these species.
